# A cross-validation scheme for machine learning algorithms in shotgun proteomics

**DOI:** 10.1186/1471-2105-13-S16-S3

**Published:** 2012-11-05

**Authors:** Viktor Granholm, William Stafford Noble, Lukas Käll

**Affiliations:** 1 Science for Life Laboratory, Department of Biochemistry and Biophysics, Stockholm University, Solna, Sweden; 2 Department of Genome Sciences, University of Washington, Seattle, USA; 3 Department of Computer Science and Engineering, University of Washington, Seattle, USA; 4 Science for Life Laboratory, School of Biotechnology, KTH - Royal Institute of Technology, Solna, Sweden

## Abstract

Peptides are routinely identified from mass spectrometry-based proteomics experiments by matching observed spectra to peptides derived from protein databases. The error rates of these identifications can be estimated by target-decoy analysis, which involves matching spectra to shuffled or reversed peptides. Besides estimating error rates, decoy searches can be used by semi-supervised machine learning algorithms to increase the number of confidently identified peptides. As for all machine learning algorithms, however, the results must be validated to avoid issues such as overfitting or biased learning, which would produce unreliable peptide identifications. Here, we discuss how the target-decoy method is employed in machine learning for shotgun proteomics, focusing on how the results can be validated by cross-validation, a frequently used validation scheme in machine learning. We also use simulated data to demonstrate the proposed cross-validation scheme's ability to detect overfitting.

## Background

Shotgun proteomics relies on liquid chromatography and tandem mass spectrometry to identify proteins in complex biological mixtures. A central step in the procedure is the inference of peptides from observed fragmentation spectra. This inference is frequently achieved by evaluating the resemblance between the experimental spectrum and a set of theoretical spectra constructed from a database of known protein sequences of the organism under consideration. If a peptide present in the database was analyzed in the mass spectrometer and triggered a fragmentation event, then the peptide can be identified by comparing the observed and theoretical fragmentation spectra. This matching procedure is carried out by search engines, such as Sequest [1], Mascot [2], X! Tandem [3] and Crux [4]. Each generated match is referred to as a *peptide-spectrum match *(PSM) and is given a score, indicating the degree of similarity between the observed and the theoretical fragmentation spectrum. The best scoring peptide of a spectrum is referred to as the spectrum's top-scoring PSM, and normally only this PSM is kept for further analysis. Here, we refer to the search engine scores as *raw scores*, because they have not been calibrated and generally lack a direct statistical interpretation. Ideally, the best raw score is assigned to the PSM of the peptide that originally produced the spectrum. Subsequently, from the set of PSMs, the proteins in the sample can be inferred [5-8].

A large proportion of the fragmentation spectra in shotgun proteomics experiments are matched to peptides that were not present in the fragmentation cell when the spectrum was collected. We say that the top-scoring PSMs for these spectra are incorrect. In practice, the researcher generally chooses a score threshold above which PSMs are deemed significant and considered to be correct matches. However, because there are many error sources associated both with the mass spectrometer and the matching procedures, correct and incorrect PSMs cannot be completely discriminated using raw scores. For this reason, an important step in the analysis is to estimate the error rate associated with a given score threshold. These error rates, quantified using statistical confidence measures, are usually expressed in terms of the false discovery rate (FDR) [9-11], the expected fraction of false positives among the PSMs that are deemed significant. The closely related *q *value [11] is defined as the minimum FDR required to deem a PSM as correct. Thus, the *q *value provides a useful statistical quantity that can be readily assigned to each PSM individually.

Target-decoy analysis is arguably the most common approach for estimating error rates in shotgun proteomics. As described later, this approach uses searches against a shuffled *decoy *database to model incorrect matches. Besides error rate estimation, the target-decoy approach has been used to increase the score discrimination between correct and incorrect PSMs using semi-supervised machine learning [12-17]. This increased discrimination is highly valuable, because it typically results in a considerably higher number of confident peptide identifications. However, as we demonstrate below, improperly implemented machine learning approaches risk seriously damaging the quality of the results and the reliability of the corresponding estimated error rates. Without proper validation protocols, strong biases, such as overfitting, that undermine the basic assumptions of the target-decoy approach, will remain undiscovered.

Here, we describe the cross-validation procedure used by Percolator [13], a semi-supervised machine learning algorithm for post-processing of shotgun proteomics experiments. The procedure accurately validates the results by keeping training and validation sets separate throughout the scoring procedure. We begin by introducing the idea of target-decoy analysis. Subsequently, we focus on how ranking of PSMs can be improved by using machine learning algorithms. Finally, we will discuss how to validate the results from machine learning algorithms to ensure reliable results. The effect of the validation is demonstrated using an example based on simulated data.

## Results and discussion

### Estimating statistical confidence using the target-decoy analysis

Frequently, results from shotgun proteomics experiment are validated using the target-decoy analysis. The procedure provides a mean to empirically estimate the error rates by additionally matching the spectra against a *decoy *database. The decoy database consists of shuffled or reversed versions of the *target *database, which includes the protein sequences of the organism under consideration. As a consequence, the decoy database is assumed to make up a list of biologically infeasible protein sequences that are not found in nature. A spectrum matched against one of these sequences is termed a decoy PSM, as opposed to a standard target PSM, and is assumed to be incorrectly matched. The idea is that the decoy PSMs make a good model of the incorrect target matches, so that the error rates can be estimated [18]. In this article we assume that the target and the decoy databases are searched separately. The other main strategy, which is not discussed here, is target-decoy competition, in which a single search is made through a combined target and decoy database [19].

To estimate the FDR corresponding to a certain score threshold with separate target-decoy searches, one first sorts all PSMs according to their score. Second, one takes all PSMs with scores equal to, or above, the threshold, and divide the number of decoy PSMs by the number of target PSMs. Third, this fraction is multiplied by the expected proportion of incorrect PSMs among all target PSMs, which can be estimated from the distribution of low-scoring matches [11,20,21]. To estimate *q *values, each PSM is assigned the lowest estimated FDR of all thresholds that includes it. With this approach, the researcher finds a score threshold that corresponds to a suitable *q *value, often 0.01 or 0.05, and uses this threshold to define the significant PSMs.

### Target-decoy approach to machine learning

Let us now turn our attention on how we may improve the separation between correct and incorrect PSMs than by ranking PSMs by the search engine's raw scores alone. Correct and incorrect PSMs may have different distributions of other features than just the search engine's raw scores. We can hence design scoring functions that combine such features and obtain better separation between correct and incorrect PSMs. The features that we want to include in such a combined scoring function can be selected from a wide set of properties of the PSMs. The features might describe the PSM itself, such as the fraction of explained b- and y-ions; the PSM's peptide, such as the peptide's length; or the PSM's spectrum, such as the spectrum's charge state.

We can use machine learning techniques, such as support vector machines (SVMs) [22], artificial neural networks, or random forests to obtain an, by some criterion, optimal separation between labeled examples of correct and incorrect PSMs. The method that we will discuss here, Percolator, uses a semi-supervised machine learning technique, self-training [23] linear SVM [24], to increase the separation between correct and incorrect PSMs. [13] Semi-supervised machine learning algorithms can use decoy PSMs and a subset of the target PSMs as examples to combine multiple features of PSMs into scores that identify more PSMs than the original raw scores.

The target-decoy analysis relies on the assumption that the decoy PSMs are good models of the incorrect target PSMs. To extend the target-decoy analysis to include the scenario where we have combined different PSM features into one scoring function, we have to assure that the used PSM features for decoy PSMs are good models of the ones of incorrect target PSMs. For many features, this assumption requires that the target and decoy databases are as similar as possible. To assure the same amino acid composition, and size, the decoy is made from the target database by shuffling [25], using Markov [26] or bag-of-word models [27] or reversing [18,19] it. Only reversing, however, promises the same level of sequence homogeneity between the two databases, as shuffling would lead to larger variation among decoy peptides than target peptides. Furthermore, to conserve the same peptide mass distribution between the two databases, the peptides are often pseudo-reversed [28]. In that case, each amino acid sequence between two enzymatic cleavage sites is reversed, while the cleavage sites themselves remain intact.

### Confounding variables

In all mass spectrometry-based proteomics experiments random variation will make full separation between correct and incorrect PSMs very hard, if not impossible, to achieve. Such variation can be introduced during the experimental procedures, but also during the subsequent bioinformatics processes. Sample concentration, instrument type and sequence database composition [29] are just a few of many elements potentially hampering the search engine's separation performance.

**Figure 1:**
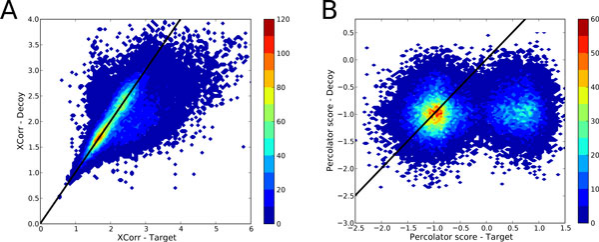
**Elimination of confounding variables in PSM scoring**. Approximately 30,000 top-scoring PSMs were obtained from a set of *C. elegans *spectra using a ± 3 Da Sequest search. A target database of *C. elegans *protein sequences and a separate decoy database of the reversed sequences were used. Each PSM obtained a target and a decoy score, indicated in the 2D-histograms on the *x *and *y *axis, respectively. The black line represents the *x *= *y *diagonal. (A) shows the score distribution of PSMs when using Sequest's XCorr. (B) shows the same PSMs when scored with Percolator score. The PSM count in each 2D-bin is indicated by color coding.

Just as in many other measurement problems, it turns out that *confounding variables *have a considerable detrimental effect on the discriminative power of a search engine [30]. Confounding variables are variables that inadvertently correlate both with a property of the PSM's spectrum or peptide, and the search engine score. Thus, the score assigned to a PSM by the search engine does not exclusively indicate the quality of the match between peptide and spectrum, but also influences from confounding variables. A typical confounding variable for *e.g*. Sequest's XCorr is the precursor ion's charge state. Single charge precursor spectra are known to have a significantly lower XCorr than multiple charged spectra. [31] Hence, the precursor charge state is a variable of the spectrum that correlates also with the search engine score. Figure 1A shows Sequest's XCorr, influenced by a covariation of properties, charge state and others, for each spectrum. The detrimental effect of this correlation between target and decoy scores for each spectrum becomes apparent when studying this figure. Some spectra obtain a high or low scores both against the target and the decoy database, regardless of their PSMs being correct or incorrect. Thresholds will inadvertently have to include some incorrect PSMs of high scoring spectra from the list of accepted target PSMs, while excluding some correct PSMs from low scoring spectra.

Removing, or decreasing, the influence of confounding variables can improve the discrimination between correct and incorrect PSMs considerably. Machine learning approaches such as PeptideProphet [32], Percolator [13] or q-ranker [16] find the most discriminating features in each particular dataset, and combine these to improve the separation. On top of rendering results with additional information from the different features taken into account, the outputted score is less influenced by confounding variables, and has better discriminative performance. As an example, the effects of using Percolator scores instead of Sequest's XCorr are shown in Figure 1B.

### Cross-validation

Regardless of whether one uses an SVM, such as Percolator, or any other machine learning approach, it is necessary to validate the performance of the algorithm. As with the common raw scores, the target-decoy approach can be applied on the scores stemming from the trained learner, to estimate the new error rates of the identifications. However, the example data used for training the algorithm is not suitable for estimating the error rates, as the training examples are likely to be, at least somewhat, overfitted.

Overfitting is a common pitfall in statistics and machine learning, in which the classifier learns from random variations in the training data. [33,34] Such learning is undesired, as it does not arise from overall trends and patterns that are generalizable to new data points. For this reason, all sound machine learning approaches keep an independent validation set separate from the training set. First, the classifier learns from the training set, to find the best scoring function. Second, the learned scoring function is applied on the validation set. This procedure helps avoid overfitting, and gives a better estimate of the performance. [35]

In shotgun proteomics, a naїve straightforward separation of the PSMs into a training set and a validation set would decrease the number of PSMs that can be outputted in the final results, as we cannot apply the learned SVM score on the set used for training. To avoid this, previous versions of Percolator employed duplicate decoy databases, one of which was used to drive the learning, and the second to apply the learned classifier on. The scores given to the PSMs by the second decoy database was used for estimating the error rates of the target PSMs. With this approach, however, the target PSMs are still used both for learning and validation, and the approach was thus removed from Percolator.

**Figure 2:**
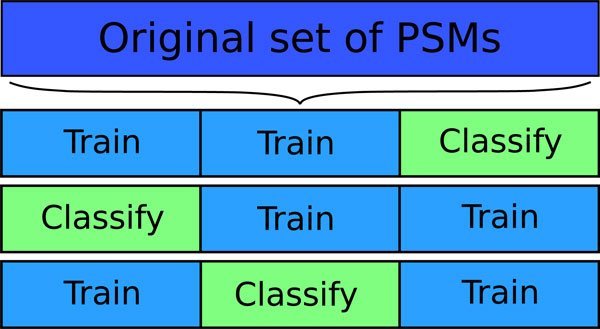
**The three-fold cross-validation procedure of Percolator**. Percolator discriminates between correct and incorrect PSMs by dividing the PSMs into three equally sized subsets. The PSMs of each subset are processed by an SVM-classifier that learned from the other two subsets.

**Figure 3:**
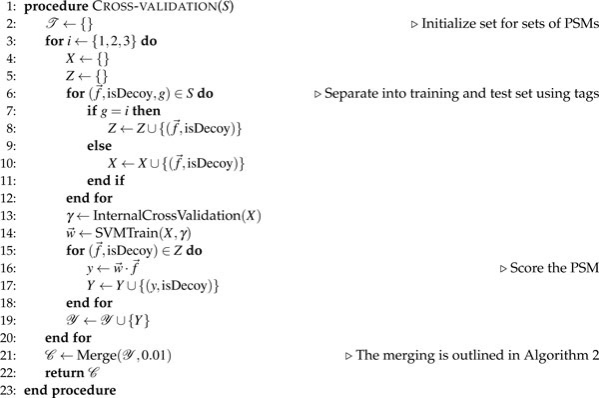
**The algorithm for cross-validation**. Given a set, *K*, of tuples ($\vec{f}$, isDecoy, *g*) representing PSMs, where $\vec{f}$ is a vector of PSM features, isDecoy is a boolean variable indicative of whether the PSM is a decoy PSM or not, and *g *∈ {1, 2, 3} is a tag indicating which cross-validation set the tuple should be allocated to, the algorithm returns a set of PSMs. The function InternalCrossValidation() is used for nested cross-validation within the training set and returns the most efficient set of learning hyperparameters. The SVMTrain() function uses the training set and hyperparameters and returns the learned feature weights needed to score the PSM.

As opposed to using duplicate decoy databases, current versions of Percolator employ cross-validation, a common method to deal with small training sets in machine learning [33,35-37]. Cross-validation means to randomly divide the input examples into a number of equally sized subsets, and to train the classifier multiple times, each time on all but one of the subsets. After each training procedure, the excluded subset is used for validation. The number of subsets can be varied, but is commonly denoted *k*. Consequently, in a *k*-fold cross-validation procedure, *k *- 1 subsets are used for training, and 1 subset for testing. This is repeated *k *times to use all possible combinations of training and validation sets. With this approach, all the data points can be classified and validated, while still keeping a separate training set. Consequently, to reliably score all PSMs, Percolator employs a three-fold cross-validation procedure by dividing the spectra into three equally sized subsets. The target and decoy PSMs from two of the subsets are used for training, and the PSMs of the spectra in the third subset for validation. The three-fold cross-validation procedure in Percolator is illustrated in Figure 2 and outlined in pseudo-code in Figure 3.

**Figure 4:**
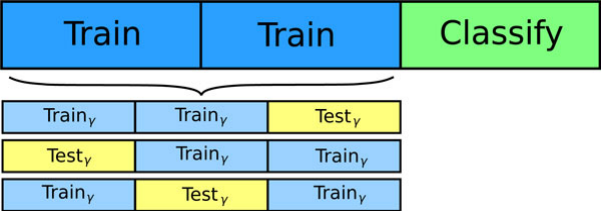
**Nested three-fold cross-validation procedure of Percolator**. Each of the three cross-validation training sets are further divided into three nested cross-validation sets, intended to select the most suitable hyperparameters for the SVM training procedure. Just as for the global cross-validation scheme, different permutations of two subsets for training and one subset for testing are used to evaluate each set of hyperparameters.

An SVM can learn from training data using different settings, or hyperparameters [38]. The best set of hyperparameters for the dataset at hand are usually approximated by a so-called grid search. This search is performed by training and validating the classifier multiple times, each time with a different permutation of hyperparameters. The hyperparameters with the best validation results is then used for the actual training. Percolator uses a nested three-fold cross-validation step within each training set to perform a grid-search. The two training subsets are divided once again into three parts, of which two at the time serve as training data, and the third as validation data. The nested cross-validation is performed for each combination of hyperparameters, so that the best combination can be chosen for training the classifier on the two top-level training sets. The nested cross-validation scheme used in Percolator is illustrated in Figure 4.

### Merging separated datasets

**Figure 5:**
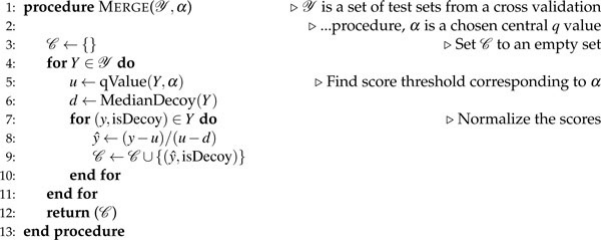
**Percolator's algorithm for normalizing the scores from different cross-validation sets**. The algorithm takes two inputs: a set  Y containing sets of PSMs, and a significance threshold *α*. Each PSM is represented as a tuple: a score and an accompanying boolean indicating whether this is a decoy PSM. The function qValue takes as input a set of scored PSMs and finds the minimal score that achieves the specified significance *α*, and MedianDecoy returns the median decoy score from the given set. The function returns a combined collection of normalized scores.

The cross-validation is necessary to prevent overfitting, but has the drawback that the three subsets of PSMs are scored by three different classifiers. These subsets cannot be directly compared, as each classifier produces a unique score learned from the features of its respective input examples. Nevertheless, for the researcher the three subsets have no experimental meaning and they must be merged into a single list of PSMs. To merge data points from multiple classifiers, they are given a normalized score, called the SVM score, based on the separation between target and decoy PSMs. In Percolator, the normalization is performed after an internal target-decoy analysis within each of the three classified subsets. The subset score corresponding to a *q *value of 0.01 is fixed to an SVM score of 0. And the median of the decoy PSM subset scores is set to -1. Both the target and the decoy PSM scores within the subset are normalized with the same linear transformation, using the above constrains. Figure 5 outlines in pseudo-code how the normalization and merging is done in practice.

After the normalization, the three subsets of PSMs are merged, and the overall error rates are estimated by target-decoy analysis on all PSMs. The final result is a list of PSMs and accurate error rates, where correct and incorrect matches have been highly discriminated.

### Other issues with validation

In the previous sections, we described a cross-validation procedure that assures that the machine learning algorithm only considers general patterns in the data, and not random variations within a finite dataset. However, the fundamental assumption that decoy PSMs are good models of incorrect target PSMs hasstill not been validated. This assumption can be validated by analyzing mixtures of known protein content, in which incorrect target PSMs are readily identified. Such validation experiments enable direct comparisons of these incorrect matches and the decoy PSMs. For machine learning algorithms, it is important to validate that each one of the features considered by the learner are indeed very similar between decoy and incorrect target PSMs. Else, the classifier would easily detect these features, and produce biased results. An example of such a feature is the number of PSMs matching to the same peptide sequence, which differs slightly between decoy and incorrect target PSMs. [39]

### Simulated example

We evaluated the ability of our cross-validation strategy to avoid overfitting by letting it train on a series of simulated datasets. Each dataset consisted of 2500 target and 2500 decoy synthetic PSMs, described by 50 randomly generated features. All random features followed a normal distribution with mean of 0.0 and standard deviation of 1.0. To 1000 of the target synthetic PSMs, we added an off set of 10.0 to the first feature, to simulate correctly matched PSMs. With this procedure, 100 datasets were created, and the performance of Percolator was tested on each one of them. To demonstrate the effects of Percolator's cross-validation scheme, we also ran Percolator with the cross-validation protocol disabled.

**Figure 6:**
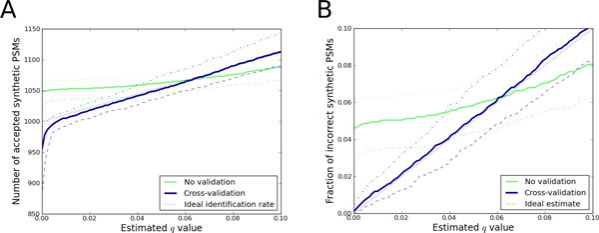
**The effect of cross-validation on simulated datasets**. Percolator was run with and without the cross-validation protocol enabled (blue and green lines, respectively) for 100 simulated datasets. Each dataset contained 2500 synthetic target and decoy PSMs, represented by 50 randomly generated features. 1000 of the target PSMs were intentionally made different, as examples of synthetic "correct" matches. The medians of the 100 runs are shown by full lines, and the lower and upper dashed lines represent the 5% and 95% quartiles. (A) shows the number of synthetic PSMs deemed significant for each *q *values threshold. (B) shows the fraction of synthetic incorrect PSMs among the accepted PSMs against the estimated *q *values.

Given that *q *represents the *q *value, the ideal identification rate from the above experiment is 1000/(1 - *q*). In other words, we hope to find 1000 PSMs with a *q *value of 0, but more when we increase the *q *value and start to introduce incorrect PSMs among the reported PSMs. As seen in Figure 6A, without cross-validation, Percolator overestimates the number of significant synthetic PSMs. With cross-validation, on the other hand, Percolator outputs results close to the ideal identification rate. Additionally, as seen in Figure 6B, cross-validation ensures that the identified synthetic PSMs are the correct ones. Without it, the estimated error rates (*q *values) are not accurate.

## Conclusions

Here, we discussed the cross-validation implementation used by Percolator to ensure the reliability of the machine learning output. With a three-fold cross-validation procedure, no data points are lost, while still keeping separate training and validation sets. The PSMs from the three resulting classifiers are merged after first normalizing the three scores, and the error rates are estimated using straightforward target-decoy analysis based on the normalized scores.

Although cross-validation is used by machine learning algorithms in all fields, merging the validated data afterwards is less common. In shotgun proteomics, normalizing scores and merging the data is a necessity, for instance to allow analyzing unique peptides, where multiple PSMs map to the same peptide sequence. Thus, a normalization procedure is a natural second step after the cross-validation. As there is no established general-purpose method to normalize the scores in the different cross-validation sets, we had to design our own heuristic procedure. As we have described here, we chose to linearly rescale the scores before merging the datasets. This procedure lacks support in the literature but seems to work well in practice.

## Competing interests

The authors declare that they have no competing interests.
